# Muscle-Tendon Unit Properties *during* Eccentric Exercise Correlate with the Creatine Kinase Response

**DOI:** 10.3389/fphys.2017.00657

**Published:** 2017-09-19

**Authors:** Kirsty M. Hicks, Gladys L. Onambele-Pearson, Keith Winwood, Christopher I. Morse

**Affiliations:** ^1^Department of Sport, Exercise & Rehabilitation, Northumbria University Newcastle Upon Tyne, United Kingdom; ^2^Department of Exercise and Sport Science, Health Exercise and Active Living Research Centre, Manchester Metropolitan University Crewe, United Kingdom; ^3^School of Healthcare Science, Manchester Metropolitan University Manchester, United Kingdom

**Keywords:** creatine kinase, exercise-induced muscle damage, fascicle strain, maximal isometric torque loss, tendon stiffness

## Abstract

**Aim:** The aim of this paper was to determine whether; (1) patella tendon stiffness, (2) the magnitude of vastus lateralis fascicle lengthening, and (3) eccentric torque correlate with markers of exercise induced muscle damage.

**Method:** Combining dynamometry and ultrasonography, patella tendon properties and vastus lateralis architectural properties were measured *pre* and *during* the first of six sets of 12 maximal voluntary eccentric knee extensions. Maximal isometric torque loss and creatine kinase activity were measured *pre-damage (*−*48 h), 48, 96, and 168* h post-damage as markers of exercise-induced muscle damage.

**Results:** A significant increase in creatine kinase (883 ± 667 UL) and a significant reduction in maximal isometric torque loss (21%) was reported post-eccentric contractions. Change in creatine kinase from pre to peak significantly correlated with the relative change in vastus lateralis fascicle length during eccentric contractions (*r* = 0.53, *p* = 0.02) and with eccentric torque (*r* = 0.50, *p* = 0.02). Additionally, creatine kinase tended to correlate with estimated patella tendon lengthening during eccentric contractions (*p* < 0.10). However, creatine kinase did not correlate with resting measures of patella tendon properties or vastus lateralis properties. Similarly, torque loss did not correlate with any patella tendon or vastus lateralis properties at rest or during eccentric contractions.

**Conclusion:** The current study demonstrates that the extent of fascicle strain during eccentric contractions correlates with the magnitude of the creatine kinase response. Although at rest, there is no relationship between patella tendon properties and markers of muscle damage; during eccentric contractions however, the patella tendon may play a role in the creatine kinase response following EIMD.

## Introduction

Although it is well accepted that unaccustomed eccentric exercise results in functional and cytoskeletal impairments, referred to as exercise-induced muscle damage (EIMD), the mechanical determinants, which govern the severity of EIMD still remains unclear. Previously, the differences in EIMD have been attributed to fascicle strain (Lieber and Friden, 1993; Peñailillo et al., 2015; Guilhem et al., 2016), eccentric force (Warren et al., 1993; Chapman et al., 2008), the elastic properties of the tendon (although not confirmed experimentally) (Marginson et al., 2005; Guilhem et al., 2016) and, in women, circulating estrogen levels (Carter et al., 2001). At present, these aforementioned determinants have predominately been identified using various *in situ* and *in vitro* conditions, with limited studies investigating the determinants *in vivo* (Chapman et al., 2008; Peñailillo et al., 2013; Hoffman et al., 2014; Guilhem et al., 2016). Although integral to the understanding of EIMD, *in vitro* experiments do not include the in series elastic components of the muscle, often involve single fibers and induce eccentric strains beyond the physiological range (Butterfield, 2010). Further insight into the mechanical processes resulting in EIMD *in vivo* is critical for health and sport practitioners to understand the metabolic and structural response to eccentric exercise programs.

Recently, the tendon has been reported to play an important role during eccentric contractions *in vivo* and has been shown to reduce fascicle lengthening (Hicks et al., 2013; Hoffman et al., 2014) and mediate peak force, peak torque and fascicle velocity *in situ* (Roberts and Azizi, 2010). Therefore, in accordance with Morgan's (1990) popping sarcomere theory, by attenuating the degree of fascicle lengthening, the tendon may limit EIMD (Hoffman et al., 2014). Although previously, Hicks et al. (2016) reported no significant correlation between resting patella tendon stiffness and the CK response, the role of tendon stiffness on functional markers of EIMD, such as torque loss, remains unknown. Interestingly, Guilhem et al. (2016) proposed that a more compliant Achilles tendon resulted in shorter fascicle lengthening and subsequently less damage. A conclusion could not be drawn however, as their experimental design did not manipulate tendon stiffness, and pooled male and female participants, the tendon properties of which are known to differ significantly (Kubo et al., 2003; Onambélé et al., 2007; Hicks et al., 2013). Therefore, to investigate whether tendon stiffness correlates with markers of EIMD, a range of tendon stiffness's need to be investigated whilst controlling for confounding variables such as sex.

Following the recent insight into the interaction between the patella tendon and fascicle lengthening during eccentric muscle contractions *in vivo* (Hicks et al., 2013), fascicle lengthening has been investigated as a crucial determinant of EIMD (Hoffman et al., 2014; Peñailillo et al., 2015; Guilhem et al., 2016). The majority of these studies however, have been conducted using submaximal, multi-joint exercise (Hoffman et al., 2014; Peñailillo et al., 2015). Multi-joint movements associated with submaximal exercise may have obscured the role of fascicle lengthening on EIMD. During single joint, maximal eccentric contractions of the plantar flexors *in vivo*, Guilhem et al. (2016) reported that maximal fascicle length was correlated with torque loss and the delayed onset of muscle soreness post EIMD. The relative contribution of fascicle lengthening to total muscle-tendon lengthening during eccentric contractions however, is significantly lower in the plantar flexors (51%, Guilhem et al., 2016) compared to the VL (89%, Hicks et al., 2013). Therefore, to further our understanding of the relationship between fascicle lengthening and EIMD, a muscle group with a higher contribution of fascicle lengthening to total muscle-tendon unit lengthening needs investigating.

In addition to high strain (fascicle lengthening), the production of high torque is another characteristic of eccentric contractions which has been investigated as a determinant of EIMD (Lieber and Friden, 1993; Warren et al., 1993; Chapman et al., 2008; Guilhem et al., 2016). Within the elbow flexors and plantar flexors both Chapman et al. (2008) and Guilhem et al. (2016) (respectively) reported no correlation between eccentric torque and markers of EIMD. Interestingly, within the plantar flexors, Guilhem et al. (2016) reported a signficant correlation between negative work performed (fascicle lengthening × eccentric torque) and torque loss, thus providing tangible evidence that eccentric torque may contribute to EIMD. The average eccentric torque reported by Guilhem et al. (2016) (100 N·m) however, is substantially lower than the average eccentric torque reported within the VL (255 N·m, Hicks et al., 2016). Therefore, futher investigation into a larger muscle group is required to gain a greater insight into the potential determinants of EIMD.

Although fascicle lengthening and eccentric torque have all been investigated as potential determinants of EIMD in muscle-tendon complexes (Hoffman et al., 2014; Peñailillo et al., 2015; Guilhem et al., 2016), the mechanical processes which predispose the degree of EIMD, specifically within the VL, remains unclear. Furthermore, due to limited research, the relationship between patella tendon properties and indirect markers of EIMD remains unknown. Therefore, the aim of this paper was to determine whether (1) *patella tendon stiffness*, (2) *the amount of VL fascicle lengthening (strain)* and (3) *eccentric torque* correlate with markers of EIMD.

## Materials and method

### Subjects

Sixteen males (21.1 ± 1.6 years of age, 72.0 ± 7.5 kg and 176 ± 6 cm) signed written informed consent to participate in this study. All participants self-reported as being recreationally active (undertaking no more than 1 h of “moderate” physical activity per week) and did not take part in any structured resistance training. All procedures complied with the Declaration of Helsinki and ethical approval was obtained through the Ethics Committee of Manchester Metropolitan University. Exclusion criteria included any resistance training in the last 6 months, occupation or lifestyle that required regular heavy lifting or carrying, any known muscle disorder, the use of dietary supplements (i.e. vitamin E), and any musculoskeletal injury in the last 3 months. All inclusion and exclusion criteria were determined via a questionnaire prior to inclusion within this study.

### Testing protocol

Once selected, participants were asked to visit the laboratory on five different occasions over a period of 9 days. Although different participants have been used, the design and several of the measurement techniques within the current study have been reported previously (Hicks et al., 2013, 2016). The sessions were as follows: (1) *pre-damage (48 h prior to damage)* (2) *damage* and (3) *48 h*, (4) *96 h*, and (5) *168 h post-damage*. *Pre-damage* assessments consisted of mass and stature (anthropometric measures), 5–6 ml blood sample, patella tendon moment arm, isokinetic-dynamometer familiarization, morphological and mechanical measures of the patella tendon (tendon size and stiffness), VL anatomical cross-sectional area (VL_ACSA_) and resting architecture, and two maximal isometric voluntary knee extension torque (MVC_KE_) measurements at 60, 65, 70, 75, 80 and 90°. Participants performed two practice MVC_KE_, at 60° and 70° during the familiarization session. Mass and stature were measured using digital scales (Seca model 873, Seca, Germany) and a wall mounted stadiometer (Harpenden, Holtain Crymych, UK) respectively. The *damage* session consisted of eccentric exercise during which the degree of fascicle lengthening was measured using ultrasound, a 5–6 mL venous blood sample and MVC_KE_ torque measurements. *48, 96, and 168 h session* consisted of 5–6 mL blood sample and MVC_KE_ torque measurements.

All tests were carried out on the self-reported non-dominant leg, which was defined as the leg that provided stability during movements which require balance e.g., kicking a ball. Participants were seated, with a hip angle of 85°, in an isokinetic-dynamometer (Cybex Norm, Cybex International, NY, USA). Participants were secured in a seated position using inextensible straps around the hips and shoulders. The isokinetic-dynamometer axis of rotation was visually aligned with the knee joint's center of rotation. The isokinetic-dynamometer settings, including the anatomical zero, were recorded during pre-damage and replicated in the following sessions.

### Vastus lateralis anatomical cross-sectional area

Using a real-time B-mode ultrasound (AU5 Harmonic, Esaote Biomedica, Genoa, Italy) VL_ACSA_ was measured. To identify 50% of VL length, the participant laid supine with their leg fully extended (knee angle 0°), the proximal and distal insertions sites of the VL were identified using an ultrasound probe (7.5 MHz linear array probe, 38 mm wide). At 50% VL length, the medial and lateral border of the VL were identified using the ultrasound. Using a fabric tape measure, axial sections were marked using echo absorptive markers every 30 mm from the medial border to the lateral border of the VL. Using the osseous surface as an alignment guide, the ultrasound probe was orientated in the axial-plane, perpendicular to the VL muscle, and steadily moved over the echo-absorptive markers from the medial to the lateral edge of the VL. Minimal pressure was applied to the ultrasound probe to avoid compression of the muscle. The images were recorded in real time at 25 frames per second (Adobe Premier pro Version 6, Adobe Systems Software, Ireland). Using capturing software (Adobe Premier Elements, version 10), individual images were acquired at each 30 mm interval. Shadows cast by the echo-absorptive markers allowed the images to be aligned by the outline of the muscle, thus forming the entire VL_ACSA_ in a single image (Adobe Photoshop Elements, version 10). Digitizing software (ImageJ 1.45, National Institutes of Health, USA) was used to measure VL_ACSA_. This method of calculating VL_ACSA_ has previously been accepted as reliable and valid when compared to MRI, with a reported interclass correlation between 0.998 and 0.999 and a coefficient of variation of 2.1% (Reeves et al., 2004).

### Patella tendon length and cross-sectional area

A real-time B-mode ultrasound (AU5 Harmonic, Esaote Biomedica, Genoa, Italy) was used to measure patella tendon cross-sectional area and patella length at a fixed 90° knee angle. The distance between the apex of the patella and the tibial tuberosity, marked using sagittal ultrasound images, was taken as patella tendon length. With the ultrasound probe orientated in the transverse plane images were captured at 25, 50, and 75% of patella length to measure patella tendon cross-sectional area. Using image analysis software, the ultrasound images were later analyzed offline (ImageJ 1.45, National Institutes of Health, USA). High reliability for measuring patella tendon length and patella tendon cross-sectional area was reported within the current study (CV 0.69 and 3.50% respectively).

### Patella tendon stiffness

The method for measuring patella tendon stiffness has been detailed previously (Hicks et al., 2013, 2016). In brief, the participants were seated in the isokinetic dynamometer, with the knee angle fixed at 90°, and were instructed to perform a ramped, isometric MVC_KE_ lasting ~5–6 s. Ultrasound images of the patella tendon and ramped MVC_KE_ torque were synchronized using a 10-V square wave signal generator. Ramped MVC_KE_ torque was presented on a Macintosh G4 computer (Apple Computer, Cupertino, CA, USA), via an A/D converter and subsequently analyzed with the accompanying software (Acknowledge, Biopac Systems, Santa Barbara, CA). Patella tendon displacement was measured over two ramped MVC_KE_, once with the probe at the distal edge of the patella and the second with the probe over the tibial tuberosity (Onambélé et al., 2007). Total patella tendon displacement was calculated as displacement at the apex of the patella plus the displacement at the tibial tuberosity (Onambélé et al., 2007). An echo-absorptive marker was placed on the skin perpendicular to the patella tendon. The marker cast a shadow on the ultrasound image delineating the position of the skin and deep tissue. The shadow acts as a fixed reference point from which, the distance from an anatomical reference point at the start of the contraction to the end of the contraction can be measured as tendon elongation. Ultrasound images were captured, and total patella tendon displacement was measured at ~10% intervals of the ramped MVC_KE_ torque output (Onambélé et al., 2007). Patella tendon forces were calculated as: (MVC_KE_ torque + antagonist co-activation torque) / patella tendon moment arm. The methods used to measure patella tendon moment arm and antagonist co-activation torque during MVC_KE_ are described in detail below.

The force—patella tendon elongation curve stemming from data at every 10% of ramped MVC_KE_ was then fitted with a second-order polynomial function forced through zero (Onambélé et al., 2007). The tangential slope at discreet sections of the curve, relative to MVC_KE_ force, was computed by differentiating the curve at every 10% force intervals. To standardize the comparison of tendon stiffness and Young's modulus at an absolute load, the slope of the tangential line, corresponding to the MVC_KE_ force of the weakest participant (2,972 N·m), was computed for each subject.

### Patella tendon moment arm

Patella tendon moment arm was measured at 90° (full extension = 0°) in the sagittal plane, from a dual-energy X-ray absorptiometry scan (frame 23.3 × 13.7 cm, Hologic Discovery, Vertec Scientific Ltd, UK), and subsequently analyzed using a DICOM image assessment tool (OsiriX DICOM viewer, ver. 4.0, Pixemo, Switzerland). Patella tendon moment arm length was determined as the perpendicular distance from the center of the patella tendon to the tibio–femoral contact point. Dual-energy X-ray absorptiometry scans have been compared to MRI measures, demonstrating consistent reliability and validity against this standard (Erskine et al., 2014).

### Antagonist co-activation torque

To determine co-activation during the ramped MVC_KE_, electromyography (EMG) of the bicep femoris was measured. Ultrasound, in the axial plane, was used to confirm that the placement of two biopolar electrodes (Ambu, Neuroline 720, Denmark) was in the mid-sagittal line at 25% of bicep femoris muscle length (distal end = 0%). To reduce skin impedance below 5,000 Ω, the skin was shaved, gently abraded and cleansed with an alcohol wipe prior to electrode placement. The electrodes were placed in a bipolar configuration with a constant inter-electrode distance of 20 mm. A reference electrode (Ambu, Blue Sensor, Denmark) was placed on the lateral tibial condyle. The raw EMG signal was amplified (×2000) and filtered (through low and high band pass filters of 10 and 500 Hz respectively), with a common mode rejection ratio of 110 dB (50 Hz) and sampling frequency of 2,000 Hz. Participants performed two maximal voluntary isometric knee flexions (MVC_KF_) at 90°. The participants were instructed to perform the contractions as rapidly and as forcefully as possible. The participants were instructed to relax once a 2 s plateau on the dynamometer screen had been observed. Ramped MVC_KE_ torque and bicep femoris EMG were recorded in real time and synchronized using a 10-V square wave signal generator. The root mean square of the bicep femoris EMG signal, was calculated 500 ms either side of instantaneous MVC_KF_ peak torque. The baseline signal noise was calculated as the root mean square over 1 s and removed from the measured electromyography during MVC_KF_ and MVC_KE_. At every 10% of ramped MVC_KE_ torque the root mean square of the bicep femoris EMG was taken over 250 ms. Therefore, using the aforementioned methods, knee flexor co-activation torque was calculated as described by Onambélé et al. (2007);

$$\text{KF Coactivation}=\left(\frac{{\text{BF}}_{\text{RMS}}{\text{MVC}}_{\text{KE}}}{{\text{BF}}_{\text{RMS}}{\text{MVC}}_{\text{KF}}}\right)\cdot {\text{MVC}}_{\text{KF }}\text{Torque}$$

Where, BF_RMS_ = root mean square of the bicep femoris EMG, MVC_KE_ = maximal isometric voluntary knee extension torque, MVC_KF_ = maximal isometric voluntary knee flexion torque.

It must be noted that several assumptions have been made for the calculation of co-activation. Firstly, it has been assumed that the bicep femoris is representative of the entire hamstring muscle group (Carolan and Cafarelli, 1992) and secondly, in accordance with previous literature (Lippold, 1952), it is assumed that the relationship between bicep femoris electromyography and MVC_KF_ torque is linear. Finally, inline with previous research (Onambélé et al., 2007), hamstring co-activation torque was calculated solely from the bicep femoris, which due to not calculating semitendinosus and semimembranosus co-activation torque, may result in an under represented total hamstring co-activation.

### Patella tendon stress/strain relationship

Patella tendon stress was calculated by dividing patella tendon force (N) by patella tendon cross-sectional area (mm^2^). Patella tendon strain (%) was calculated as the ratio between total patella tendon displacement to patella tendon length.

### Young's modulus

Young's modulus was calculated by dividing patella tendon length (mm) by patella tendon cross-sectional area (mm^2^), then multiplying the answer by patella tendon stiffness.

### Maximal isometric voluntary knee extensor torque measurements

At six different knee angles (60, 65, 70, 75, 80, and 90° (full extension = 0°)) participants were instructed to perform two MVC_KE_ lasting ~2 s with 90 s rest between contractions. Torque was presented, in real time, on a Macintosh G4 computer (Apple Computer, Cupertino, CA, USA), via an A/D converter (Biopac Systems, Santa Barbara, CA). Torque measurements were later analyzed offline with the accompanying software (Acknowledge, version 3.9.2). The highest torque produced at each angle was taken as MVC_KE_ peak torque. During *pre-damage* the angle at which the highest MVC_KE_ torque was produced was recorded as optimal knee angle. To calculate loss of MVC_KE_ torque following eccentric exercise, MVC_KE_ were repeated at the knee angle determined as optimal during *pre-damage*, 1-h post eccentric exercise (to reduce any fatigue effect) and *48, 96*, and *168 h* post eccentric exercise.

### “Damaging” eccentric exercise

Prior to eccentric exercise, a warm-up of four isokinetic concentric knee extensions and knee flexions were carried out, ensuring a progressive increase in effort (with the last contraction being maximal). For the eccentric exercise, the knee extension range of motion was set at 20–90° (0° = full extension). Participants performed 12 maximal eccentric voluntary knee extensions (MVE_KE_) repetitions at 30°·s^−1^, for six sets. During the concentric phase the leg was passively returned to 20° at an angular velocity of 60°s^−1^. Participants had to remain seated during their 2 min rest between each set. Verbal encouragement and visual feedback was continuously provided throughout the protocol. MVE_KE_ torque measurements were later analyzed offline. For each set, peak MVE_KE_ torque was determined as the highest torque out of the 12 repetitions.

### Change in vastus lateralis fascicle length during the eccentric protocol

To measure VL fascicle length during MVE_KE_, the ultrasound probe (7.5 MHz linear array probe, 38 mm wide) was held in position by the experimenter at 50% of VL muscle length in the mid-sagittal plane of the non-dominant leg. To provide a visual reference point for the internal structures, an echo-absorptive marker was fixed onto the skin at 50% of VL muscle length. When measuring fascicle lengthening from 20° to 90° knee angle during MVE_KE_, pilot data reported the probe to move a negligible 0.02 ± 0.05 cm proximally, and therefore was not considered further in calculations for the present study. VL fascicle length was determined as the linear distance along the fascicle as it ran from the deep to the superficial aponeurosis. A hypo-allergenic ultrasound gel (Parker, Park Laboratories Inc., Fairfield, UK) was used to enhance acoustic coupling between the skin and the ultrasound probe.

During the first set (out of six) of MVE_KE_ contractions, ultrasound images were recorded onto a PC, in real time, at 25 frames per second (Adobe Premier pro Version 6). Only the first set was analyzed due to Guilhem et al. (2016) reporting no significant effect of eccentric set number on fascicle lengthening. A 10-V square wave signal generator was used to synchronize the ultrasound images with the torque acquisition. Three MVE_KE_ contractions were chosen at random from the first set of 12 repetitions for architectural analysis. Using frame capture software (Adobe Premier Elements, version 10) the ultrasound image corresponding to every 10°, from 20° to 90° was acquired for offline analysis. Movement of the shadow casted by the echo-absorptive marker would act as an indicator that the probe had moved during the MVE_KE_ therefore if any movement was observed, the contraction was discarded and another repetition was chosen for analysis.

Using digitizing software (ImageJ 1.45, National Institutes of Health, USA), VL fascicle length was analyzed offline at every 10°. Fascicle length was measured from the visible insertion of the fiber from the deep into the superficial aponeurosis (Reeves and Narici, 2003). Where the fascicle extended longer than the ultrasound image (frame width 3.50 cm and height 4.15 cm), linear continuation of the fascicle and aponeurosis was assumed. Within the VL, a 2–7% error is associated with the linear extrapolation method when used to calculate VL fascicle length (e.g., measured at 11.3 cm) at a knee angle of 120° (Finni et al., 2003). Furthermore, using a 40 and 38 mm probe width respectively, Guilhem et al. (2011) and Hicks et al. (2013) both report high reliability when measuring VL fascicle lengthening during MVE_KE_. In agreement with previous research, the current study reported high reliability when measuring VL fascicle lengthening during MVE_KE_ (ICC 0.99 and CV 2.95%). In order to reduce any error associated with the estimation of VL fascicle length, an average of three fascicles across the image was taken (Guilhem et al., 2011). Fascicle length during eccentric contractions was measured at every 10° knee angle (range 20°–90°, 0° = full extension) throughout the MVE_KE_. Change in fascicle length is presented as fascicle length at a knee angle of 90° made relative to fascicle length measured at a knee angle of 20°; hereafter termed “relative fascicle lengthening” and reported as a percentage change from starting length at 20°.

### Vastus lateralis total muscle-tendon unit excursion

In order to estimate the total VL muscle-tendon unit elongation, the tendon excursion method was adopted (Spoor et al., 1990); whereby the patella tendon moment arm at 90° knee angle was multiplied by the change in knee angle (70°, 1.22 rad) during the MVE_KE_.

### Blood samples

To measure CK levels a 21-gauge needle was inserted into the antecubital vein of the forearm, and 5–6 mL of blood was drawn into a serum collection tube. The sample was allowed to clot whilst on crushed ice for 60 min and then centrifuged at 4,500 rpm at 0°C for 10 min. Using a 200–1,000 μl pipette (Eppendorf, USA), the resulting serum sample was separated into three aliquots (~500 μl each) and stored in Eppendorf tubes at −20°C until later CK analysis. Creatine kinase activity was measured using colorimetry at 340 nm optical density (BioTek EL × 800 96 well Microplate Reader), with enzyme activity calculations carried out using a generic software (Gen5, version 2.0). Each sample was run in duplicate using an EnzyChromTM CK Assay Kit (BioAssay Systems, Hayward, CA, sensitivity 5 U/L, intra-assay variability <5%, data from the manufacturer). An average of the two readings was taken as the enzyme activity at each experimental phase. Throughout this manuscript CK activity is reported in two ways: absolute values and peak CK above baseline values (i.e., the change from pre to peak CK over the 168 h (ΔCK_peak_)).

### Statistics

The statistical software package SPSS (v.19, Chicago, IL) for Windows and Microsoft Excel were used to run statistical analysis. To check for parametricity, the Levene's and Shapiro-Wilk tests were used to assess the variance and normality of the data respectively. A one way repeated measures ANOVA (time, 5 levels) was used for CK and MVC_KE_ torque loss. The greenhouse-Geisser correction factor was applied if the assumption of sphericity was violated. If a significant main effect was reported, a pairwise comparison, with a bonferroni correction, was used to identify which time point was significantly different to pre-damage. A one way repeated measures ANOVA (sets, 6 levels) was used to investigate MVE_KE_ torque during the EIMD protocol. *T*-tests were used to compare the increase from pre, to peak CK (ΔCK_peak_) and pre, to to peak MVC_KE_ torque loss. Linear correlations (Pearson *r*) were used to determine whether ΔCK_peak_ or absolute and relative MVC_KE_ torque loss, correlated with either muscle properties, tendon properties, fascicle lengthening or MVE_KE_ torque. Data is presented as mean ± standard deviation. Statistical significance was accepted at *p* < 0.05.

## Results

### Pre-damage vastus lateralis and patella tendon properties

**Figure 1:**
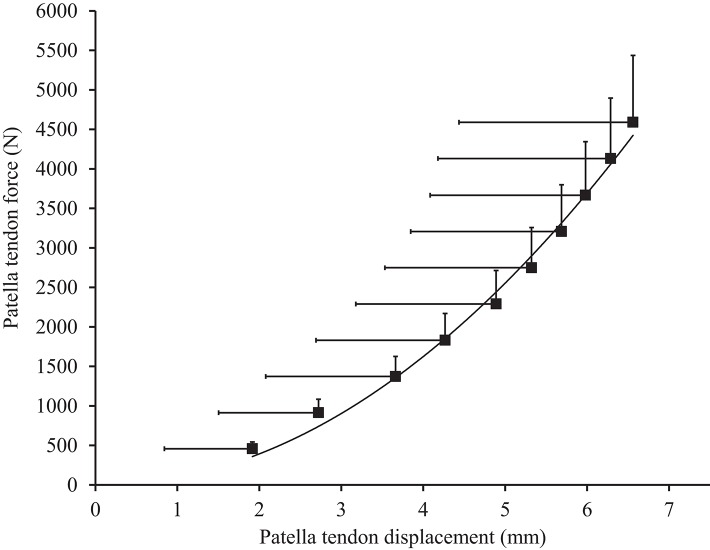
Patella tendon force-displacement relationship in males. Values are presented as mean ± standard deviation.

Muscle architecture and tendon properties, assessed at the “pre-damage phase,” are presented in Table 1. Resting patella tendon length and patella tendon cross-sectional area was 57.8 ± 6.0 mm and 78.1 ± 26.1 mm^2^ respectively. Patella tendon moment arm at 90°' knee angle was 4.33 ± 0.34 cm. To account for varying maximal MVC_KE_ torque during ramped MVC_KE_ (195.6 ± 36.8 N·m), the patella tendon force (2,972 N) corresponding to the highest MVC_KE_ torque (127.2 N·m) of the weakest participant was used to calculate standardized force level patella tendon stiffness (1,213 ± 436 N·mm^−1^, Figure 1) and Young's modulus (1,030 ± 591 MPa).

**Table 1:** Correlations between VL and patella tendon properties during rest and EIMD with ΔCK_peak_ and absolute and relative MVC_KE_ torque loss.

	**Mean ± SD**	**ΔCK_peak_ (U/L)**		**MVC**_**KE**_ **torque loss (NM)**		**MVC**_**KE**_ **torque loss (%)**	
		**Pearson' *r***	***p*-value**	**Pearson' *r***	***p*-value**	**Pearson' *r***	***p*-value**
**RESTING MEASURES**							
VL muscle length (cm)	32.8 ± 2.1	0.28	0.15	0.11	0.35	−0.06	0.41
VL_ACSA_ (cm^2^)	25.3 ± 4.2	0.36	0.09^a^	0.22	0.21	0.16	0.27
Fascicle length (20°)	7.1 ± 0.4	−0.21	0.23	0.16	0.27	0.01	0.49
Maximal tendon stiffness (N·mm^−1^)	1, 450 ± 554	0.33	0.11	−0.29	0.14	−0.27	0.16
Maximal Youngs's modulus (MPa)	1, 065 ± 511	0.22	0.21	−0.19	0.24	−0.18	0.25
Relative tendon stiffness (N·mm^−1^)	1, 214 ± 436	0.13	0.31	−0.29	0.14	−0.22	0.21
Relative Young's modulus (MPa)	890 ± 394	0.07	0.39	−0.19	0.24	−0.15	0.29
**DURING EIMD**							
Tendon elongation (cm)	1.1 ± 1.2	−0.41	0.06^a^	−0.35	0.09^a^	−0.22	0.21
Average MVE_KE_ (Nm)	255 ± 51	0.50	0.02^*^	−0.02	0.47	−0.20	0.23
Change in fascicle length (cm)	4.2 ± 0.9	0.41	0.06^a^	0.33	0.10	0.19	0.24
Relative change in fascicle length (%)	59.4 ± 12.0	0.53	0.02^*^	0.29	0.14	0.18	0.25
Negative work	0.6 ± 0.2	0.50	0.03^*^	0.07	0.39	0.02	0.47

*MVC_KE_, Maximal voluntary isometric knee extension; ΔCK_peak_, Increase from pre CK to peak CK over the 168 h post EIMD; MVE_KE_, Maximal voluntary eccentric knee extension*.

* *Denotes a significant correlation (p < 0.05). ^a^Denotes a correlation trend (p < 0.10)*.

### Maximal eccentric voluntary knee extension torque during the eccentric protocol

MVE_KE_ torque was not significantly different throughout the six sets (*p* = 0.868). During EIMD, average MVE_KE_ peak torque (calculated over the six sets) was 255 ± 51 N·m. Peak MVE_KE_ torque was 97 ± 16% of “pre-damage” MVC_KE_ torque.

### Vastus lateralis fascicle lengthening during eccentric protocol

A significant change in fascicle length from 20° knee angle (7.06 ± 0.43 cm) to 90° knee angle (11.3 ± 0.20 cm) was seen (4.20 ± 0.82 cm, *p* = 0.0004) during MVE_KE_. The change in VL fascicle length relative to fascicle length at knee angle of 20° was equivalent to a 59.4 ± 12.0% increase in fascicle lengthening during MVE_KE_.

### Estimated vastus lateralis excursion

Based on the tendon moment arm excursion, the estimated increase in VL muscle-tendon unit length from 20 to 90° knee angle was 5.29 ± 0.41 cm during MVE_KE_.

### Creatine kinase levels

Creatine kinase significantly increased from pre-damage to 96 h (136 ± 114 U/L, 796 ± 723 U/L respectively, *p* = 0.014) but there was no significant difference at 1 (430 ± 104 U/L, *p* = 0.167), 48 (425 ± 82 U/L, *p* = 0.051) and 168 h (281 ± 58, *p* = 0.774) post EIMD.

**Figure 2:**
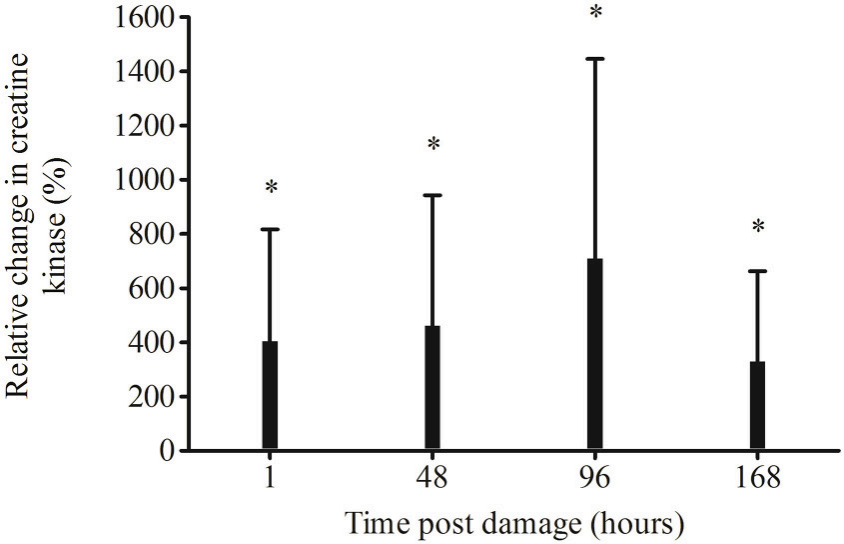
Relative change in creatine kinase from pre-damage (−48 h), following exercise-induced muscle damage. ^*^Significantly higher than pre-damage *p* < 0.01.

Compared to pre-damage, relative CK was significantly higher at every time point post EIMD (1 *p* = 0.004, 48 *p* = 0.004, 96 *p* = 0.002 and 168 h *p* = 0.007, Figure 2). ΔCK_peak_ (peak CK value—the pre CK values) was 883 ± 667 UL equating to an 885% increase in CK from pre-damage.

### Maximal isometric voluntary knee extensor torque loss

**Figure 3:**
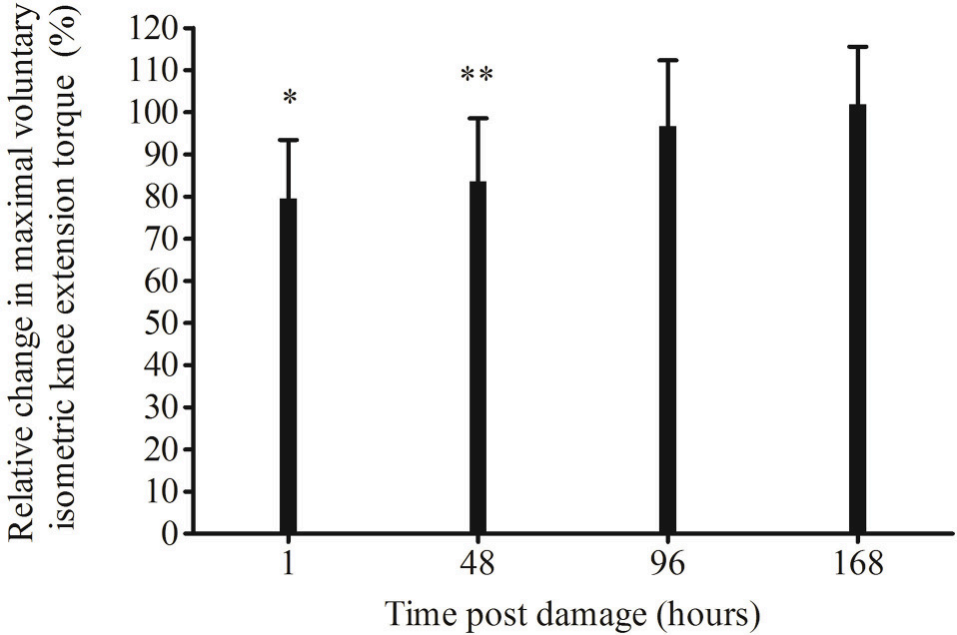
Relative change in maximal voluntary isometric knee extension torque following exercise-induced muscle damage. Significantly lower than pre ^*^p < 0.001, ^**^p < 0.01.

MVC_KE_ torque significantly decreased from pre-damage (264 ± 35 N·m), 1 h (209 ± 42 N·m, *p* = 0.0004) and 48 h (221.0 ± 48.4 N·m, *p* = 0.004) post EIMD, but had returned to pre-damage by 96 (256 ± 14 N·m, *p* = 1.00) and 168 h post damage (270 ± 13 N·m, *p* = 1.00). When made relative to pre-damage, a significant reduction in MVC_KE_ torque loss remained 1 h (*p* = 0.0004) and 48 h (*p* = 0.005) post EIMD, but was not significantly different at any other time point (Figure 3). There was a significant rightward shift in the optimal MVC_KE_ knee angle, from pre-damage to post EIMD (mean, 77 ± 9°, and 85 ± 7°, respectively, *p* = 0.002).

### Correlations between markers of exercise-induced muscle damage

Linear correlations between the markers of muscle damage and VL and tendon patella tendon properties *pre-damage* and during EIMD are presented in Table 1.

**Figure 4:**
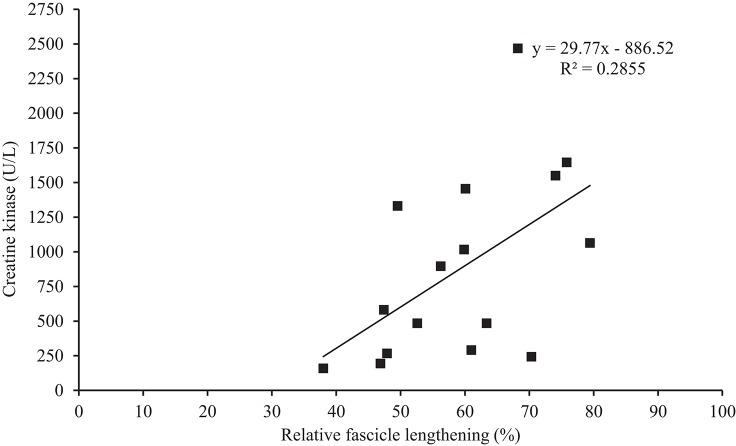
Correlation between creatine kinase (from pre to peak creatine kinase over the 168 h (ΔCK_peak_)) and relative change in fascicle length.

ΔCK_peak_ did not correlate with any resting patella tendon or VL properties (Table 1). During EIMD, ΔCK_peak_ demonstrated a correlation trend with change in fascicle length, however when fascicle length was made relative to fascicle length at 20° knee angle a significant correlation was identified (Figure 4). Additionally, during EIMD, a correlation trend (*p* < 0.10) was reported between ΔCK_peak_ and estimated tendon lengthening. Finally, ΔCK_peak_ significantly correlated with MVE_KE_ torque and negative work (relative fascicle lengthening multiplied my MVE_KE_ torque).

MVC_KE_ torque loss did not correlate with any resting patella tendon or VL properties (Table 1). During EIMD MVC_KE_ torque loss displayed a correlation trend with estimated tendon elongation however, no significant correlation was reported with any patella tendon or VL properties. When made relative to pre-damage MVC_KE_ torque loss did not correlate with any resting patella tendon or VL properties (Table 1).

## Discussion

The aim of the current study was to determine whether (1) *patella tendon stiffness*, (2) *the amount of VL fascicle lengthening (strain)*, and (3) *eccentric torque* correlate with markers of EIMD. The current study reports three main findings; (1) During EIMD, VL relative fascicle lengthening, MVE_KE_ torque and negative work correlated significantly with ΔCK_peak_, (2) Patella tendon properties did not correlate with ΔCK_peak_ or MVC_KE_ torque loss. (3) There was no significant correlations reported with MVC_KE_ torque loss. Within the current study, the VL was considered a surrogate of the quadriceps. Although there is currently no measure to quantify the individual muscle damage within the quadriceps, previous studies have reported VL to be a reliable surrogate for predicting force output for the quadriceps (Alkner et al., 2000; Moreau et al., 2010).

### Patella tendon stiffness

Following previous observations of muscle-tendon interactions during eccentric contractions (Roberts and Azizi, 2010; Hicks et al., 2013), it has been suggested that tendon properties may contribute to the magnitude of EIMD (Hicks et al., 2013; Peñailillo et al., 2015; Guilhem et al., 2016). For example, a more compliant tendon (Patella and Achilles respectively) has been reported to attenuate fascicle lengthening and reduce peak forces and torques during eccentric contractions (Hicks et al., 2013; Roberts and Konow, 2013). Despite the tendon gaining a reputation as a “mechanical buffer” during EIMD, in agreement with our previous work (Hicks et al., 2013), the current study reported no significant correlation between patella tendon stiffness and ΔCK_peak_. In addition we report no significant relationship between patella tendon properties and MVC_KE_ torque loss (Table 1). Our current findings therefore suggest that patella tendon mechanical characteristic do not correlate with markers of EIMD within the VL. Further research into tendons displaying different mechanical characteristics compared to the patella tendon, for example the Achilles tendon, is required to support the findings of the current study.

### Patella tendon lengthening during EIMD

In-line with previous research, patella tendon stiffness was measured during an isometric contraction (Reeves et al., 2003; Onambélé et al., 2007); however, tendon stiffness may only be meaningful if it is measured *during* the eccentric contraction. Therefore, to estimate the contribution of the patella tendon during the eccentric protocol within the current study, the total excursion of the VL muscle-tendon unit was estimated using the tendon excursion method (Spoor et al., 1990). In brief, based on the measured patella tendon moment arm at a 90° knee angle and a 70° change in knee angle during MVE_KE_ (equivalent to the change in knee angle during the eccentric contraction from 20 to 90°), the total estimated muscle-tendon unit lengthening was 5.29 ± 0.41 cm. Therefore, by subtracting the measured degree of VL fascicle lengthening (4.20 ± 0.82 cm) during the eccentric protocol from the estimated total muscle-tendon unit lengthening, patella tendon lengthening can be estimated as 1.09 cm. Therefore, using the aforementioned equation, estimated patella tendon lengthening during the eccentric protocol demonstrated a correlation trend with both MVC_KE_ torque loss and ΔCK_peak_, such that patella tendon lengthening can explain (taken from the *r*^2^) ~12% and ~17% of MVC_KE_ torque loss and ΔCK_peak_ within the current study. In agreement with the current study, despite relative Achilles tendon lengthening being greater than patella tendon lengthening (1.99 and 1.09 cm respectively), Guilhem et al. (2016) reported a correlation trend between Achilles tendon lengthening during eccentric contractions and MVC torque loss. These findings highlight that although tendon stiffness properties measured during an isometric contraction do not correlate with markers of EIMD, the degree of patella tendon lengthening *during* the eccentric contraction may be more important.

### Fascicle lengthening during exercise induced muscle damage

In accordance with the popping sarcomere theory, the degree of fascicle lengthening has previously been investigated as a determinant of EIMD (Lieber and Friden, 1993; Peñailillo et al., 2015; Guilhem et al., 2016; Hicks et al., 2016). The present study shows *in vivo* that the relative change in VL fascicle length during eccentric loading is significantly correlated with ΔCK_peak_. It must be noted however, that CK is an indirect marker of EIMD and it is difficult to determine whether an increase in CK represents a change in cell membrane permeability or structural damage (Heled et al., 2007).

Contrary to previous research investigating the plantar flexors (Guilhem et al., 2016), the current study did not find a significant correlation between relative VL fascicle lengthening and MVC_KE_ torque loss. These discrepancies occurred despite the contribution of relative fascicle lengthening to total muscle-tendon unit lengthening being greater within the VL compared to the plantar flexors [4.20 cm (79%) and 2.31 cm (51%) Guilhem et al., 2016, respectively]. It is accepted that EIMD is significantly higher at longer compared to shorter muscle lengths (Newham et al., 1988), the aforementioned discrepancies may be attributed to the current study limiting fascicle lengthening to a 90° knee angle, due to the constraints of safely performing MVE_KE_, whereas within the plantar flexors EIMD was performed closer to the plantar flexors end range of motion (Guilhem et al., 2016). Therefore, future studies need to measure fascicle lengthening through a volitional range of motion at the knee, to determine if total fascicle lengthening correlates with MVC_KE_ torque loss.

Within the current study, a 38 mm probe was used to measure fascicle lengthening during the eccentric contractions. Fascicle length at 90° reached nearly three times the probe length thus resulted in a large proportion of the fascicle being estimated by the linear extrapolation method. Although, previous research (Guilhem et al., 2011; Hicks et al., 2013) that used similar probe lengths to the current study, have concluded this method as reliable, it must be recognized that future studies may require a wider probe (50 mm) to reduce the estimated proportion of the fascicle during eccentric contractions.

### Eccentric torque during EIMD

In addition to strain, it has previously been reported that eccentric torque is a determinant of EIMD (Nosaka and Sakamoto, 2001). In agreement with previous *in vivo* research (Chapman et al., 2008; Guilhem et al., 2016) the current study reported no direct correlation between MVE_KE_ torque and MVC_KE_ torque loss. It must be noted that MVE_KE_ torque made relative to *pre-damage* MVC_KE_ torque within the current study and previous studies (Chapman et al., 2008; Guilhem et al., 2016) (97, ~77, and 94% respectively) is below the suggested yield strength of muscle fibers as identified within animal studies (>113%, Warren et al., 1993). Therefore, the insignificant correlation between MVE_KE_ torque and MVC_KE_ torque loss within the current and previous research (Chapman et al., 2008; Guilhem et al., 2016) may be attributed to low MVE_KE_ torque to MVC_KE_ torque ratio. In the present study, this low ratio of eccentric to isometric torque, would be expected given the low lengthening velocity used (30°·s^−1^; Onambele et al., 2004). It could be suggested that within the current study participants were not contracting maximally (even at these lower eccentric speeds), using electrical stimulation to elicit higher eccentric torques may have altered the recruitment pattern (Crameri et al., 2007) and potentially masked any protective mechanism of the muscle on EIMD. Therefore, by not using electrical stimulation, the current study has reported data using an eccentric torque achieved during voluntary maximal effort. Additionally, it must be acknowledged that due to short recovery between repetitions within the current study, the occurrence of fatigue during the eccentric protocol may have reduced the MVE_KE_ torque to MVC_KE_ torque ratio. Although it is difficult to separate the occurrence of fatigue and EIMD, due to no significant reduction in MVE_KE_ torque from set one to set six, the current study can be confident that fatigue did not affect MVE_KE_ torque production.

Although independently fascicle lengthening and MVE_KE_ torque did not correlate with MVC_KE_ torque loss within the current study, Guilhem et al. (2016) reported a stronger significant correlation when negative work, which encompassed fascicle lengthening beyond slack length multiplied by MVE_KE_ torque, was calculated. Within the current study however there was no significant correlation between negative work (relative fascicle lengthening multiplied my MVE_KE_ torque, Table 1) and MVC_KE_ torque loss. However, further investigation into higher MVE_KE_ torque to MVC_KE_ torque ratios and a full volitional range of motion at the knee to elicit maximal fascicle lengthening is required.

To the authors' knowledge, the current study is the first study to report a significant correlation between MVE_KE_ torque and ΔCK_peak_. Furthermore, unsurprisingly, due to CK demonstrating a significant relationship with both relative fascicle lengthening and MVE_KE_ torque, calculating negative work performed (relative fascicle length multiplied by relative MVE_KE_ torque, Table 1) significantly correlated with CK. Although the CK response post EIMD is accepted as an indirect marker of muscle damage, it is unclear whether CK is a true representation of muscle function and the magnitude of damage or whether it reflects a change in the cell membranes permeability to intramuscular proteins (Friden and Lieber, 2001; Heled et al., 2007). Therefore, the significant correlation between CK and both fascicle lengthening and MVE_KE_ torque within the current study, may support a relationship between cell membrane permeability and the occurrence of EIMD, rather than the quantitative severity of EIMD *per se*. Additionally, no significant correlation between MVC_KE_ torque loss, which is regarded as a structural and functional marker of EIMD (Clarkson and Hubal, 2002), and fascicle lengthening or MVE_KE_ torque further supports the notion that CK may be representative as a qualitative measure of EIMD. Future studies are required to use direct marker of muscle damage (e.g., biopsies and Z-line streaming) or a non-invasive alternative (e.g., elastography Yanagisawa et al., 2015, length-tension relationship Hoffman et al., 2014) to determine whether fascicle lengthening is a determinant of structural EIMD. Furthermore, due to a MVE_KE_ torque correlating with CK and not MVC_KE_ torque loss, despite low MVE_KE_ torque, suggests that CK may be sensitive to lower MVE_KE_ torques. Therefore, with the caveats associated with CK in mind, to confirm whether MVE_KE_ torque correlates with MVC_KE_ torque loss and thus EIMD, greater MVE_KE_ torques or smaller muscle groups need to be investigated.

## Conclusion

To conclude, the current study highlights the potential mechanistic role of tendinous and fascicle lengthening during eccentric contractions and the influence these factors may have on markers of EIMD. To fully understand the determinants of EIMD however, direct measurement of muscle-tendon unit properties *during* eccentric contractions, within muscles which display significantly higher level of EIMD compared to the VL and Plantar flexors (Guilhem et al., 2016; e.g., Bicep Femoris, Chen et al., 2011), is required. Furthermore, studies exerting a MVE_KE_ torque to MVC_KE_ torque ratio greater than ~113% may be required in order to fully understand whether MVE_KE_ torque correlates with EIMD.

## Author contributions

KH: Data collection, data analysis, and completed the write up of the manuscript. KW, GO, and CM, all contributed to data collection, data analysis and write up of the manuscript equally.

### Conflict of interest statement

The authors declare that the research was conducted in the absence of any commercial or financial relationships that could be construed as a potential conflict of interest.

## Abbreviations

ANOVA: Analysis of variance
CK: Creatine kinase
EMG: Electromyography
EIMD: Exercise-induced muscle damage
MVE_KE_: Maximal voluntary eccentric knee extensions
MVC_KE_: Maximal voluntary isometric knee extension
MVC_KF_: Maximal voluntary isometric knee flexions
OCP: Oral contraceptive pill
ΔCK_peak_: The increase from pre to peak CK over the 168 h
VL: Vastus lateralis
VL_ACSA_: Vastus lateralis anatomical cross-sectional area.
